# The Mechanism of Aerobic Exercise Regulating the PI3K/Akt-mTOR Signaling Pathway Intervenes in Hippocampal Neuronal Apoptosis in Vascular Dementia Rats

**DOI:** 10.3390/ijerph20031893

**Published:** 2023-01-19

**Authors:** Lei Gao, Fushun Liu, Ruilian Liu

**Affiliations:** 1 Department of Physical Education, Yuzhang Normal University, Nanchang 330103, China; 2 Police Sports Department, Zhejiang Police College, Hangzhou 310053, China; 3 College of Physical Education, Yichun University, Yichun 336000, China

**Keywords:** vascular dementia, aerobic exercise, PI3K/Akt-mTOR, autophagy

## Abstract

Background: The purpose of this paper is to explore the mechanism of aerobic exercise regulating autophagy through the PI3K/Akt-mTOR signaling pathway and its participation in apoptosis, to protect the hippocampal nerves from damage in vascular dementia rats. Methods: Thirty-six healthy male SD rats were randomly divided into a sham group, a model group, and a model exercise group. A neurobehavioral assessment was used to determine the memory and exploration abilities of the rats. A TUNEL assay was used to detect hippocampal neuron apoptosis. Immunohistochemical and Western blot analyses were used to analyze LC3Ⅱ and the beclin-1 protein. An RT-PCR detected the differential expression of mRNA. Results: The results of the neurobehavioral tests showed that the platform latency time of the rats with vascular dementia was prolonged. Aerobic exercise significantly shortens the swimming time of rats in platform latency. The TUNEL results showed that the TUNEL-positive cells of the hippocampal neurons in the model group increased; the expression of pro-apoptotic genes caspase-3 and Bax mRNA was up-regulated, and the expression of Bcl-2 mRNA was down-regulated. Aerobic exercise reduced hippocampal neuronal apoptosis, up-regulated Bcl-2 mRNA, and down-regulated caspase-3 and Bax mRNA. The LC3Ⅱ and Beclin-1 proteins, detected by immunohistochemistry and a Western blot analysis, showed that the protein expression in the hippocampi of rats with vascular dementia increased. Aerobic exercise reduced LC3Ⅱ and Beclin-1 protein expression. The results of the RT-PCR showed similar changes. Conclusions: Aerobic exercise could improve the learning and memory abilities of vascular dementia rats, moderately regulate the process of autophagy, reduce the TUNEL-positive cells of hippocampal neurons, repair damaged hippocampal neurons by regulating the autophagy signaling pathway PI3K/Akt-mTOR, and improve hippocampal function.

## 1. Introduction

Severe cognitive impairment syndrome with altered memory, cognition, and behavior, caused by ischemic and hemorrhagic cerebrovascular diseases, which have been induced by hypertension, diabetes, atrial fibrillation, etc., and its risk factors, are collectively referred to as vascular dementia (VD). It is the most common form of dementia after Alzheimer’s disease [1]. Clinically, VD often shows a significant decline in memory, cognitive function, thinking and judgment abilities, calculation ability, and social life ability, and it is accompanied by an abnormal personality, etc. [2]. At this stage, there is still a lack of specific therapeutic drugs and most active measures can only delay the progression of the disease and reduce functional degradation, and then achieve the effect of prevention and intervention. Autophagy refers to the catabolic process in which damaged organelles, long-lived proteins, pathogens, and other cytoplasmic components are transported to the lysosomes for degradation [3]. Autophagy can induce the transportation of these components from intracellular protein denaturation, and apoptosis, as well as induce the transportation of the damaged organelles to the lysosomes for degradation through growth, differentiation, and structural remodeling. Autophagy can also induce the clearance of cellular waste [4,5], which is beneficial to maintain homeostasis and to protect the cells from harm [6]. Although recent studies have shown that moderate autophagy has neuroprotective effects in neurodegenerative diseases, there is still a lot of controversy about the regulatory role of the autophagy intensity in CNS degeneration and vascular-factor-related cognitive dysfunction [7,8]. The degeneration of VD hippocampal neurons often involves both apoptosis and autophagy. Whether systematic aerobic exercise can improve the apoptosis of hippocampal neurons in VD and increase autophagy, has rarely been reported. Therefore, we intended to further elucidate the mechanism of inhibiting the apoptosis of hippocampal neurons by regulating autophagy and observing the changes in the expression levels of autophagy-related genes in the hippocampal neurons of VD through aerobic exercise intervention.

## 2. Materials and Methods

### 2.1. Animals and Handling

Male Sprague Dawley rats (12 months old, *n* = 36) were procured from Shanghai Slack Laboratory Animal Co., Ltd. (Shanghai, China). The animals were stabilized for seven days before starting the experiments. The rats were divided into a sham group (Sham), a vascular dementia model group (VG), and a model exercise group (VE), using a random number method, with 12 animals in each group.

### 2.2. Establishment of the Vascular Dementia Model

To generate a rat model of VD, we established a permanent bilateral common carotid artery occlusion (2-VO) approach, as has been previously reported [9,10]. The rats were fasted overnight, deprived of water for 4-h, and anesthetized with sodium pentobarbital (40 mg/kg). A neck ventral midline incision was made, the skin was separated and the related tissue was separated with microsurgical scissors and ophthalmic forceps, in order to bypass the vagus nerve, and then the surrounding tissue was separated. The common carotid artery was exposed, and the bilateral common carotid arteries were permanently ligated with surgical thread to create a model of VD. The carotids were occluded with a 1-week interval between the interventions; the right common carotid artery was the first to be processed and the left one was occluded 1 week later. The Sham rats underwent the same procedures without carotid artery ligation. All of them had their skin sutured through an anti-inflammatory method with penicillin, and they were sent to a sterile, ventilated, and air-conditioned room for 1 week.

### 2.3. Aerobic Exercise Program

The VE rats were modified by the training program which referred to the exercise load of experimental animals by Bedford [11]. The VE rats were trained for 1 week, according to the load adaptive training of a 0° gradient 10 m/min·10 min, increasing to a 10° gradient 16 m/min·60 min, and then they were exercised for 8 weeks, according to this training intensity, 6 days per week, and the training time was uniformly arranged from 9:00 to 10:00. All of the VE rats successfully completed the running tasks every day.

### 2.4. Neurobehavioral Assessment

All of the rats completed a Morris water maze test after the intervention, and their cognitive, learning, and memory abilities were assessed. The escape latency and times of crossing the platform within 2 min were recorded by the positioning cruise. Spatial exploration was used to detect the swimming time in the quadrant of the rats after removing the platform.

### 2.5. Hippocampal Tissue Sampling

All rats were fasted overnight and anesthetized with 2% sodium pentobarbital (40 mg/kg) intraperitoneally. Six rats in each group were randomly selected, their skulls were cut from the foramen magnum, and their parietal bones were removed. The whole brain tissue was exposed, the cerebral cortex was turned upward with ophthalmic forceps, to expose the hippocampus. The brain tissue surrounding the hippocampus was separated with a glass minute needle, the hippocampus was removed, blotted dry with filter paper, weighed, and the left and right sides were separated. Total RNA was extracted, and the right hippocampus was frozen at −80 °C. The remaining 6 rats had their parietal bone removed according to the above method, and the whole brain tissue was taken out and fixed in 4% paraformaldehyde for more than 48 h. The tissues were fixed, embedded in paraffin, sliced to a 5-µm thickness, and then used for a TUNEL assay and immunohistochemical detection.

### 2.6. Immunohistochemical Detection of the LC3II Protein Expression

The paraffin sections were routinely dewaxed, rehydrated, soaked in distilled water, dripped in a 3% hydrogen peroxide solution diluted with methanol, incubated at room temperature for 15 min, immersed in 0.01 M citric acid buffer, and then heated in a microwave oven at 98 °C for 22 min to repair. The sections were then washed with PBS 3 times, treated with rabbit anti-mouse LC3Ⅱ (1:1000, Abcam, Cambridge, UK), and stored overnight in a refrigerator at 4 °C. Then, the next day, the sections were washed 3 times with PBS, treated with goat anti-rabbit IgG/HRP polymer, placed in a humidified oven at 37 °C for 40 min, washed 3 more times with PBS, then developed with DAB for 10 min, and terminated with tap water. Once completed, the sections were dehydrated with gradient alcohol, made transparent with xylene, and sealed with neutral resin. Of the hippocampus tissue, 6 fields (×400) were randomly selected for each section to count the positive cells under high magnification and calculate the average value.

### 2.7. Apoptosis Detection of Hippocampal Neurons with TUNEL

The apoptosis of hippocampal neurons was detected with a TUNEL assay, according to the manufacturer’s instructions. The paraffin-embedded tissues were deparaffinized in xylene and rehydrated with ethanol series (absolute, 95%, 90%, 80%, and 70%, diluted in double distilled water). Following this and all subsequent stages, the sections were washed with PBS. The sections were treated with proteinase K (20 µg/mL in 10 mM Tris/HCI, pH 7.6) for 30 min, dripped with the TUNEL 50µL reaction mixture, and incubated in a dark room at 37 °C for 30 min in a wet box. Then, the sections were washed with PBS 3 times, 5 min/time, 100 µL of a DAB substrate was added dropwise, incubated at 25 °C for 10 min, rinsed with PBS 3 times, 5 min/time, counterstained with hematoxylin, dehydrated, made transparent, mounted with neutral resin, and randomly selected 5 visual fields under the light microscope, counting the positively stained brown nuclei which were apoptotic cells. The sections were viewed by light microscopy and only those cells with positive TUNEL staining and of apoptotic morphology were considered apoptotic.

### 2.8. RT-PCR Detection of the Related Gene Expression

According to the instructions of the total RNA extraction kit, total RNA was rapidly extracted from the frozen hippocampus tissue, and detected with OD260 and OD280 by an ultraviolet-visible spectrophotometer, to determine the concentration and purity. Using standard total RNA as a template, transcription was reversed to synthesize cDNA under the action of reverse transcriptase. The synthesis of the relevant primers was provided by Sangon Biotech (Shanghai, China) Co., Ltd. The primer sequences are shown in Table 1. Amplification occurred according to the kit instructions. The total reaction system was 30 µL with 3 replicate wells per sample. The reaction conditions were 95 °C for 5 min pre-denaturation, 95 °C for 5 s denaturation, 60 °C for 20 s annealing, 72 °C for 40 s extension, 40 cycles with each cycle extension at the end of collecting the fluorescent signal, drawing the amplification curve, and calculating the Ct value. The mRNA expression differences between groups were calculated according to the 2^−ΔΔCt^ method, and GAPDH was used as the internal reference gene.

### 2.9. Western Blot

Once the frozen hippocampal tissues were thawed, 10 μL RIPA was added to 1 mg of tissue, protease inhibitor cocktail, and PMSF, and then homogenized at a low speed with an electric homogenizer on ice until the tissue was completely lysed, the total protein was extracted and centrifuged at 12,000 r/min at 4 °C for 5 min, and this supernatant was removed. Protein quantification was tested with a BCA protein kit, then with SDS-PAGE gel, by sample loading, electrophoresis, mold transfer, Ponceau red to detect the presence or absence of bands, and TBST washing. Skimmed milk powder (5%) was blocked at room temperature for 2 h, and LC3Ⅱ and Beclin-1 (1:1000, Abcam, Cambridge, UK) antibodies were added dropwise overnight. The membrane was washed 3 times with TBST, 10 min/time, incubated with the secondary antibody on a shaker at room temperature for 2 h, washed with TBST again, identified by ECL chemiluminescence in the dark room, and then immersed in developer solution for 2 min and fixer solution for 2 min. ImageJ analyzed the gray value of the specific bands and compared the gray value of the target protein with β-actin as an internal reference.

### 2.10. Statistical Analysis

All experimental data were analyzed using SPSS 24.0 and expressed as $\bar{X}\;\pm \;S$D deviation. One-way ANOVA was used for comparisons between multiple groups, and an LSD-*t* test was used for the pairwise comparison. *p* < 0.05 was considered a statistically significant difference.

## 3. Results

### 3.1. Neurobehavioral Assessment

Compared with the Sham rats, the VD rats had a significantly longer escape latency before finding the platform (*t* = −12.886, *p* < 0.01), and the number of crossing platforms was significantly reduced (*t* = 8.200, *p* < 0.01). Compared with the VG rats, the escape latency of the rats was significantly shortened, and the number of crossing platforms was significantly increased after aerobic exercise intervention (*t* = 9.372, *t* = −3.464, *p* < 0.01). The swimming time was recorded when the animals were in the quadrant that previously contained the platform. The swimming time of the VD rats was significantly shortened in the quadrant of the platform, while the swimming time for the VE rats was significantly prolonged when the platform was removed (Table 2).

### 3.2. The Results of Apoptosis Detection of Hippocampal Neurons

Compared with the Sham rats, the TUNEL-positive cells of the hippocampal neurons in the VD rats were significantly increased (*t* = −6.960, *p* < 0.01), the expression of the pro-apoptotic genes caspase-3 and Bax mRNA was significantly up-regulated (*t* = −8.721, *t* = −19.433, *p* < 0.01), the expression of anti-apoptotic gene Bcl-2 was down-regulated (*t* = 8.448, *p* < 0.01), and the ratio of Bcl-2/Bax decreased significantly. The TUNEL-positive cells of the hippocampal neurons in the VE rats was significantly reduced (*t* = 5.726, *p* < 0.01, the expression level of the pro-apoptotic gene caspase-3 mRNA was significantly down-regulated (*t* = 4.010, *p* < 0.05), the expression level of the anti-apoptotic gene Bcl-2 was significantly up-regulated (*t* = −6.128, *p* < 0.01), and the ratio of Bcl-2/Bax was significantly increased, compared with the VG rats (*p* < 0.01) (Table 3).

### 3.3. Expression of the LC3Ⅱ Protein in the Hippocampus

**Figure 1:**
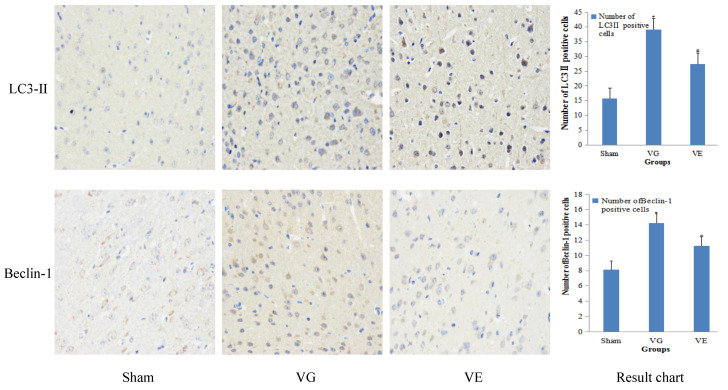
The expression level of the LC3Ⅱ protein in the hippocampus.(×400). Note: * *p* < 0.01, VS Sham, ^#^ *p* < 0.05, VS VG.

Immunohistochemical results showed that the higher the antigen content, the higher the distribution density, and the stronger the color of the positive results. According to the color rendering degree of the positive markers, blue was negative, and brown was positive. The immunohistochemistry results showed that the number of LC3Ⅱ and Beclin-1 positive cells in the hippocampus of the rats in the Sham group were few, and the cells were lightly stained. Compared with the Sham group, the number of LC3Ⅱ and Beclin-1 positive cells in the hippocampus of the VD rats increased significantly (*p* < 0.01) and showed a significant difference because the coloring was darker and brown. It can be seen that the immunohistochemical results of the VG group were more positive generally, which also made the VG group look darker than the Sham group. The number of positive LC3Ⅱ and Beclin-1 cells in the hippocampus of the VE rats was significantly decreased (*p* < 0.05), and the staining of the positive cells was significantly weakened, compared with the VG group (Figure 1).

### 3.4. Expression Results of the PI3K/Akt/mTOR Pathway and Its Related Factors

As shown in Table 4, the mRNA expression of PI3K (*t* = 6.866, *p* < 0.01), Akt (*t* = 3.628, *p* < 0.05), and mTOR (*t* = 2.796, *p* < 0.05) in the hippocampus of the rats in the VG group, were significantly down-regulated, compared with the Sham group, while LC3Ⅱ mRNA was up-regulated significantly (*p* < 0.01). Compared with the VG group, the mRNA expression of PI3K, Akt, and mTOR in the VE group, were significantly increased, showing a very significant difference (*t* = −8.966, −4.118, −14.711, *p* < 0.01), and the expression level of LC3II mRNA was significantly down-regulated (*p* < 0.01). Beclin-1, which was related with autophagy and apoptosis, had only a small amount of basal expression in the hippocampus of the rats in the Sham group, and the expression of the VG group rats was significantly increased (*p* < 0.01). The expression of Beclin-1 was significantly down-regulated in the VE group (*p* < 0.01).

### 3.5. The Results of the Western Blot

**Figure 2:**
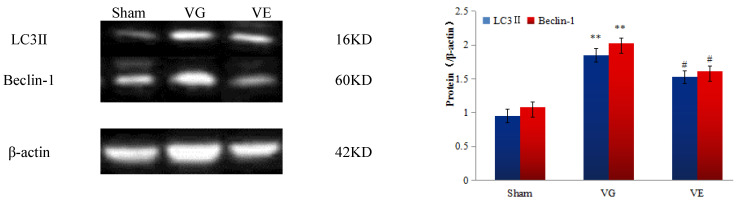
Western blot results of LC3Ⅱ and Beclin-1 in the hippocampus of the rats in each group. Note: ** *p* < 0.01, VS Sham, ^#^ *p* < 0.05, VS VG.

The results showed that only a small amount of autophagy-related proteins LC3II and Beclin-1 were expressed in the hippocampus of the Sham group, as shown in Figure 2, and in the VG group, the expression levels were significantly higher than in the Sham group (t_LC3II_ = −7.889, t_Beclin-1_ = −5.037, *p* < 0.01). The protein expression levels of LC3Ⅱ and Beclin-1 in the VE group, were significantly decreased (*p* < 0.05).

## 4. Discussion

The Morris water maze test is one of the most objective methods for evaluating the cognitive ability of experimental animals in neuropathological experiments [12,13]. Our results show that the escape latency of the vascular dementia group, to find the platform, was significantly prolonged, and the numbers for those crossing the platform was significantly reduced, while aerobic exercise shortened the escape latency time and increased the numbers for those crossing the platform. For the vascular dementia rats, when the platform was removed, the swimming time in the quadrant was shortened, and aerobic exercise prolonged the swimming time in the quadrant in the area where the platform was located. Aerobic exercise effectively improved the cognitive, learning, and memory functions of the vascular dementia rats.

Studies show that autophagy is closely related with apoptosis [14]. On the one hand, both promote each other. For example, the anti-apoptotic gene Bcl-2 interacts with the autophagy marker gene Beclin-1, which together participate in the activation of autophagy and apoptosis [15]. On the other hand, both are antagonistic to each other; autophagy can degrade damaged mitochondria to a certain extent, down-regulate the apoptosis-inducing factors, and inhibit the occurrence and development of apoptosis [16]. Nerve cell apoptosis is an important manifestation of the pathological changes of vascular dementia, especially in recent years, with evidence of apoptosis being induced by endoplasmic reticulum stress lesions. The endoplasmic reticulum stress response activates endonuclease degeneration, causes abnormal protein folding that activates the apoptosis signaling pathway, induces the occurrence and development of neuronal apoptosis, and aggravates the lesions of vascular dementia [17]. According to the TUNEL test results, the TUNEL-positive cells of hippocampal neurons in the vascular dementia rats was significantly increased, a bilateral common carotid artery ligation could accelerate the apoptosis of hippocampal neurons in vascular dementia rats; the TUNEL-positive cells of the VE rats was significantly reduced, which suggests that aerobic exercise improved the occurrence and development of hippocampal neuronal apoptosis in the vascular dementia rats, thereby improving the hippocampal neurological function. The detection results of caspase-3, Bcl-2, and Bax mRNA also confirmed this phenomenon. This might be related to the increased expression of Bax in the hippocampus of vascular dementia rats, the oligomerization in the nuclear membrane of nerve cells that affected the permeability of the nuclear membrane, increased the entry of the cytochrome into the cytoplasm, and activated the cascade of caspase-3, while the polymerization of Bcl-2 and Bax into a dimer, inhibited the production of Bcl-2, and thereby broke the balance between Bcl-2 and Bax, and induced neuronal apoptosis [18]. Aerobic exercise could promote the body’s metabolism, inhibit the oligomerization of the nuclear membrane Bax, reduce the cascade reaction of caspase-3, promote the balance between Bcl-2 and Bax, and protect the hippocampus from stress injury. The anti-apoptotic factor Bcl-2 played an important role in regulating Beclin-1 to regulate autophagy, and studies have reported that the proper inhibition of autophagy could protect cells from damage [19].

Studies have shown that the expression level of the microtubule-associated protein LC3II was proportional to the level of autophagosomes formed by nerve cells in the brain [20]. When nerve cells underwent autophagy, the expression level of LC3II was significantly up-regulated. It could be seen that LC3II, which was a specific marker factor for the detection of autophagy activity, was closely related to the degree of autophagosome formation [21].

We confirmed that the expression of Bcl-2 was down-regulated in the hippocampus of vascular dementia rats, while LC3Ⅱ and Beclin-1 were significantly increased. The down-regulation of Bcl-2 could affect the excessive autophagy regulated by Beclin-1 through the high expression of Bax. It further aggravated the apoptosis of the hippocampal neurons, which was similar to the findings of Nahid et al. [22]. This might be related to their common molecular inducers and similar regulatory mechanisms. The Bcl-2/Beclin-1 complex could be used as an important indicator for evaluating the level of autophagy [23]. Apoptosis has an important regulatory role. When vascular dementia occurs, both autophagy and apoptosis are involved in the pathological process of hippocampal neurons and jointly maintain cell homeostasis. However, when autophagy cannot maintain the homeostasis of hippocampal neurons, apoptosis dominates and induces the development of vascular dementia [24]. Beclin-1, known as an autophagy and apoptosis regulatory molecular switch protein, is an autophagy-related protein in the process of autophagosome formation, which promotes autophagosome formation. While abnormally expressed Beclin-1 activates the excessive autophagy process, it could also induce the occurrence of apoptosis [25]. Therefore, a moderate level of autophagy is beneficial to prevent apoptosis caused by excessive autophagy. As shown in the experimental results, Beclin-1 in rat hippocampal neurons was down-regulated after exercise intervention, and it maintained an appropriate level of autophagy, improved the homeostasis of autophagy and apoptosis, and blocked the apoptosis of hippocampal neurons induced by a secondary nerve injury. This suggests that aerobic exercise has a protective effect on nerve cells. This is consistent with Ding S. et al.’s report that the reduced level of Bcl-2/Beclin-1 complex could effectively protect the nerve cells in rats with a cerebral ischemia-reperfusion injury [26]. The immunohistochemistry results further verified the above conclusions, that the number of LC3II and Beclin-1 positive cells in the hippocampus of the rats in the Sham group were few, and the cells were lightly colored, which shows that there was only a low level of autophagy expressed in the hippocampus under normal conditions. The number of LC3Ⅱ and Beclin-1 positive cells in the hippocampus of the vascular dementia rats increased significantly, and the staining of the positive cells was darker. The expression of LC3Ⅱ in the hippocampus of the rats showed a very significant and continuous decrease, and the staining of the positive cells became lighter after aerobic exercise intervention. It was suggested that aerobic exercise could improve the autophagy level in the hippocampus of vascular dementia rats [27]. The results of the Western blot also showed that the expression of the autophagy-related proteins LC3Ⅱ and Beclin-1 in the hippocampus of vascular dementia rats were significantly increased, and the expression decreased after exercise intervention.

This regulatory process might be related to the differential expression of the aerobic exercise-mediated autophagy-related signaling pathways [28]. The PI3K/Akt-mTOR signaling pathway could regulate the occurrence and development of various diseases by releasing pro-survival signals, and play an important role in the process of self-repair. Previous studies confirmed that PI3K, as an important signaling factor in cells, and its downstream factors Akt and mTOR, constituted a typical pro-survival and anti-apoptotic signaling pathway, and activated mTOR to regulate autophagy [29,30]. The results of our study showed that the mRNA expression of PI3K, Akt, and mTOR in the hippocampus of the vascular dementia rats, were significantly decreased. The mRNA expression of PI3K, Akt, and mTOR were significantly increased after aerobic exercise intervention. This suggests that aerobic exercise could further activate the PI3K/Akt-mTOR signaling pathway, thereby protecting nerve cells, enhancing autophagy, and exerting an anti-apoptotic effect.

## 5. Conclusions

Bilateral common carotid artery ligation could effectively replicate the rat model of vascular dementia. In addition, it could induce a decline in the cognitive function, learning, and memory abilities of the rats and could induce an increase in the expression of pro-apoptosis-related factors, as well as a decrease in the expression of anti-apoptosis factors and the expression of autophagy-related factors, and a significant increase in the number of positive cells. Aerobic exercise intervention could moderately regulate the autophagy process, effectively reduce the apoptosis of hippocampal neurons, and protect hippocampal neural function. This process was closely related to aerobic exercise regulating the autophagy signaling pathway PI3K/Akt-mTOR, to improve hippocampal nerve apoptosis in vascular dementia rats.

## Figures and Tables

**Table 1:** Gene primer sequence.

Gene	Primer Sequence	Length (bp)
Caspase-3	Sense primer 5’-GTACAGAGCTGGACTGCGGTATTG-3’ Anti-sense primer 5’-AGTCGGCCTCCACTGGTATCTTC-3’	84
Bcl-2	Sense primer 5’-CGGGAGAACAGGGTATGA-3’ Anti-sense primer 5’-CAGAGACAGCCAGGAGAA-3’	631
Bax	Sense primer 5’-AGGGTTTCATTCCAGGATCGAGC-3’ Anti-sense primer 5’-AGGCGGTGAGGACTCCAGCC-3’	468
Beclin-1	Sense primer 5’-GCTCAGTACCAGCGAGA-3’ Anti-sense primer 5’-ACAGTACAACGGCAACTC	381
PI3K	Sense primer 5’-GAGGGGCTACGAGTGGGATA-3’ Anti-sense primer 5’-CAGGCTGGAAGGAGAAGATG-3’	81
Akt	Sense primer 5’-AGGACCCTACACAGAGGCT-3’ Anti-sense primer 5’-ACACGATGTTGGCAAAGAA-3’	81
mTOR	Sense primer 5’-AAGAAGGTCACTGAGGATT-3’ Anti-sense primer 5’-GGAGATAGAACGGAAGAAG-3’	81
LC3Ⅱ	Sense primer 5’-CGGGTTGAGGAGACACACAA-3’ Anti-sense primer 5’-ATGAGCCGGACATCTTCCAC-3’	220
GAPDH	Sense primer 5’-GTTACCAGGGTTTCCCGT-3’ Anti-sense primer 5’-GATGGTGATGGGTTTCCCGT-3’	177

**Table 2:** The results of the Morris water maze test (*n* = 12).

Group	Latency (s)	Crossing Frequency	Swimming Time ^a^ (%)
Sham	49.500 ± 5.161	5.917 ± 1.379	44.833 ± 8.632
VG	93.583 ± 12.206 **	2.500 ± 1.446 **	26.750 ± 6.905 *
VE	57.417 ± 8.017 *^,##^	4.500 ± 1.446 *^,##^	41.833 ± 10.983 ^#^

Note: ** *p* < 0.01, * *p* < 0.05, VS Sham, ^#^ *p* < 0.05, ^##^ *p* < 0.01, VS VG; ^a^: swimming time: swimming time referred to the proportion of swimming time of the rats in the quadrant of the platform.

**Table 3:** Detection of apoptosis of the neurons in the hippocampus (*n* = 6).

Group	TUNEL-Positive Cell Rates (%)	Caspase-3	Bcl-2	Bax
Sham	17.667 ± 2.658	0.582 ± 0.092	0.827 ± 0.118	1.283 ± 0.157
VG	46.500 ± 8.313 **	0.951 ± 0.053 **	0.359 ± 0.061 **	2.213 ± 0.192 **
VE	23.500 ± 4.416 *^,##^	0.825 ± 0.057 ^##^	0.645 ± 0.072 *^,##^	1.575 ± 0.131 *^,##^

Note: ** *p* < 0.01, * *p* < 0.05, VS Sham, ^##^ *p* < 0.01, VS VG.

**Table 4:** The RT-PCR detection results of the hippocampus of vascular dementia rats with aerobic exercise intervention (*n* = 6).

Group	PI3K	Akt	mTOR	LC3Ⅱ	Beclin-1
Sham	1.601 ± 0.136	1.710 ± 0.110	2.013 ± 0.042	0.166 ± 0.039	0.301 ± 0.067
VG	0.962 ± 0.116 **	1.413 ± 0.127 *	1.621 ± 0.059	0.749 ± 0.078 **	0.752 ± 0.133 **
VE	1.546 ± 0.121 ^##^	1.830 ± 0.182 ^##^	3.086 ± 0.230 ^##^	0.219 ± 0.024 ^##^	0.468 ± 0.159 ^##^

Note: ** *p* < 0.01, * *p* < 0.05, VS Sham, ^##^ *p* < 0.01, VS VG.

## Data Availability

The authors confirm that the data supporting the findings of this study are available within the article.
